# Human Trace Amine-Associated Receptor TAAR5 Can Be Activated by Trimethylamine

**DOI:** 10.1371/journal.pone.0054950

**Published:** 2013-02-05

**Authors:** Ivonne Wallrabenstein, Jonas Kuklan, Lea Weber, Sandra Zborala, Markus Werner, Janine Altmüller, Christian Becker, Anna Schmidt, Hanns Hatt, Thomas Hummel, Günter Gisselmann

**Affiliations:** 1 Department of Cell Physiology, Ruhr-University Bochum, Bochum, Germany; 2 Smell and Taste Clinic, Department of Otorhinolaryngology, University of Dresden Medical School (“Technische Universität Dresden”), Dresden, Germany; 3 Cologne Center for Genomics (CCG), University of Cologne, Cologne, Germany; Duke University, United States of America

## Abstract

In addition to the canonical olfactory receptors, TAARs were currently suggested to be a second class of chemosensory receptors in the olfactory epithelium of vertebrates. In contrast to several deorphanized murine TAARs, agonists for the intact human TAAR genes 2, 5, 6, 8 and 9 that are potentially expressed in the human olfactory epithelium have not been determined so far. Moreover, the physiological relevance of TAARs still remains elusive. We present the first successful functional expression of a human TAAR and agonists of human TAAR5. We performed a ligand screening using recombinantly expressed human TAAR5 in HANA3A cells and *Xenopus laevis* oocytes. In order to measure receptor activity, we used a cAMP-dependent reporter gene assay and two-electrode voltage clamp technique. As a result, human TAAR5 can be activated in a concentration-dependent manner by trimethylamine and with less efficacy by dimethylethylamine. It could neither be activated by any other of the tested single amines with a related chemical structure (42 in total), nor by any of the tested odorant mixtures. The hypothesis that Single Nucleotide Polymorphisms (SNP) within the reading frame of an olfactory receptor gene can cause a specific anosmia, formed the basis for clarifying the question, if anosmia for trimethylamine is caused by a SNP in a TAAR coding sequence. All functional human TAAR gene reading frames of subjects with specific anosmia for trimethylamine were amplified and products analyzed regarding SNP distribution. We demonstrated that the observed specific anosmia for trimethylamine is not correlated with a SNP in the coding sequence of one of the putatively functional human TAAR genes.

## Introduction

Trace amine-associated receptors (TAAR) belong to the family of G-protein coupled receptors (GPCR) whose first deorphanized member TAAR1, responds to biogenic trace amines like ß-phenylethylamine, p-tyramine or octopamine. Human and murine TAAR1 (h/mTAAR1) are expressed in a variety of tissues including brain, stomach, kidney, lung and intestine, but not in the olfactory epithelium (OE) [1]. In contrast to h/mTAAR1, the “olfactory TAARs” mTAAR2-9 were exclusively expressed in small subsets of olfactory sensory neurons (OSNs) in the OE [2]. Recently, TAARs have been identified as olfactory receptors (ORs) in vertebrates, because recombinantly expressed “olfactory TAARs” respond to volatile amines, amongst others N-methylpiperidine (mTAAR7f), trimethylamine (TMA) (mTAAR5) and isoamylamine (mTAAR3) [2], [3], [4]. Rat TAAR8c and 9 respond to amine extracts from urine and mTAAR4 responds to ß-phenylethylamine [5]. Other “olfactory TAARs” from rodents are still not deorphanized [6]. The mTAAR agonists TMA and isoamylamine are enriched in male mouse urine and may act as murine pheromones [7], [8]. The mTAAR4 agonist ß-phenylethylamine acts as kairomone in the chemical detection of carnivore odor by prey and is also related to stress response in both rodents and humans [5], [9], [10]. Therefore, TAARs have been suggested to be involved in the detection of social cues [2], [4], [5].

Stäubert et al. characterized the response profile of TAARs with focus on primate receptors [11]. They found out that ligands for the murine TAAR3–5 failed to activate the respective primate TAARs and assumed that TAAR receptors have lost their olfactory function in primates. In a study describing TAAR expression in the human OE, Carcinelli et al. detected mainly hTAAR5, to a lesser extend hTAAR8 and at lower levels all other hTAAR genes as well. With the exception of hTAAR1, all human TAAR cDNAs were detected exclusively in OMP-positive nasal biopsies, an indication for a specific OE expression [12].

At the present state, no ligand for a human “olfactory TAAR” receptor is known. To clarify the functionality and their role for human olfaction we performed a ligand screening for hTAAR5, the TAAR subtype with the highest expression level in human OE [12].

Human TAAR5 was functionally expressed in two different recombinant systems, HANA3A cells and *Xenopus laevis* oocytes, and screened with a panel of potentially volatile amine agonists.

## Results

### Luciferase Reporter Assay System

**Figure 1:**
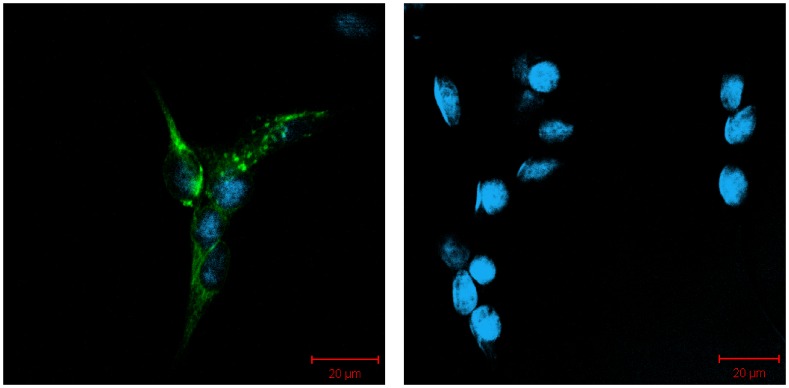
Detection of the hTAAR5 receptor protein. Expression of the rhodopsin-tagged hTAAR5 receptor in transfected, fixed HANA3A cells was detected by the anti-rhodopsin antibody 4D2 and a secondary antibody labeled with the fluorescent dye Alexa Fluor 488 (green). Cell nuclei were stained by DAPI (blue). Left: Cells transfected with hTAAR5, right: mock-transfected control cells. Scaling bar: 20 µm.

In order to find ligands for hTAAR5 we employed a Cre-luciferase reporter gene assay in which TAAR receptor activation leads to an elevation of cAMP and the subsequent expression of luciferase as described for odorant receptors [13]. We transfected HANA3A cells with cDNA coding for a rho-tagged hTAAR5 together with G_olf_ and RTP1S to ensure functional expression of the receptor, and the cAMP dependent reporter gene construct Cre-luciferase [14]. Presence of the expressed receptor protein was tested by immunocytochemical detection of the extracellular N-terminal rho-epitope tag in fixed HANA3A cells (Fig. 1) and by live-cell staining on the surface of HANA3A cells (Figure S1).

**Figure 2:**
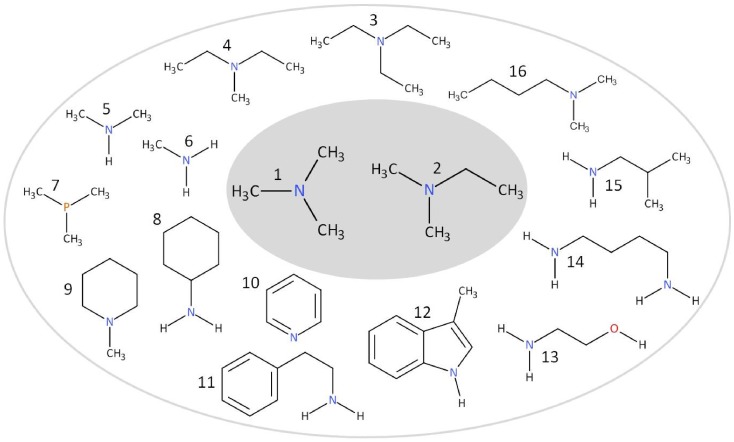
Chemical structure of various tested TMA analogs. Only tertiary amines (1) trimethylamine and (2) dimethylethylamine can activate hTAAR5. (3) triethylamine, (4) diethylmethylamine, (5) dimethylamine, (6) methylamine, (7) trimethylphosphine, (8) cyclohexylamine, (9) N-methylpiperidine, (10) pyridine, (11) β-phenylethylamine, (12) skatole, (13) ethanolamine, (14) putrescine, (15) isobutylamine, (16) dimethylbutylamine.

**Figure 3:**
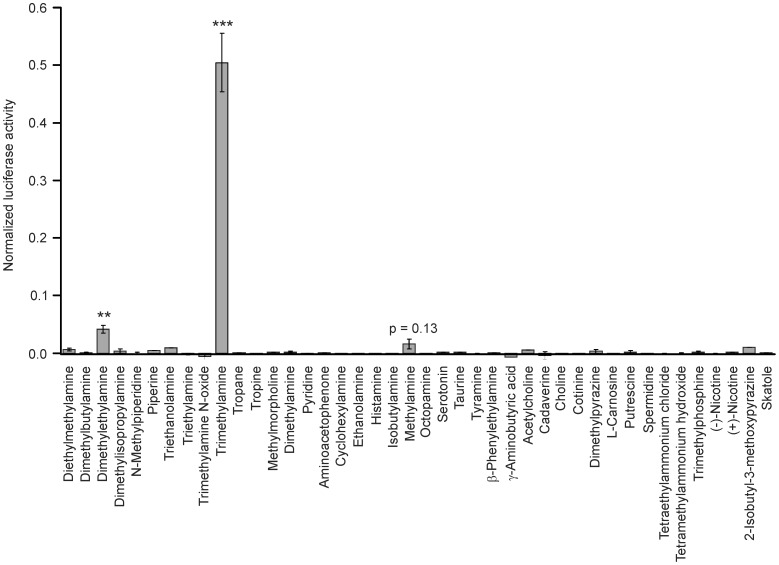
Human TAAR5 is selectively activated by TMA and DMEA. Responses of hTAAR5 to 42 different amines or amine-like substances. The concentration of all tested substances was 100 µM. Responses were normalized to the response to forskolin (10 µM). Data are given as mean ± SEM of 2–10 independent experiments, each performed in duplicates. TMA and DMEA induced signals significantly differing from mock-transfected controls (***p<0.001; **p<0.01).

In total, 42 amines or amine related substances were tested for an agonistic action on hTAAR5 (Materials and methods). Transfected cells were stimulated by 100 µM of the test substances for 4 h and induced luciferase activity was assayed by luminescence activity. Tested repertoire (Materials and methods) first based on the volatile amines known as agonist for the murine “olfactory TAARs” [2] and trace amine ligands for h/mTAAR1 [1], [15]. After finding out that hTAAR5 can be activated by TMA, we further tested chemical analogs of TMA. The chemical variations range from primary amines (methylamine) to quaternary amines (tetramethylammonium hydroxide) or diamines (putrescine) as well as to the substitution of nitrogen by phosphate or the substitutions of methyl groups by longer aliphatic chains, which form acyclic-, cyclic-, heterocyclic- or aromatic amines (Fig. 2). All chemical variants tested, abolished the activity on the hTAAR5 receptor as shown by the lack of signal increase (Fig. 3). Furthermore, the natural occurring trimethylamine N-oxide was inactive in concentrations up to 1 mM (data not shown). Only the tertiary amines TMA (100 µM) and the substitution from one methyl group by one ethyl group, namely dimethylethylamine (DMEA) (100 µM), significantly (p<0.001) activated hTAAR5 expressing cells (Fig. 3).

**Figure 4:**
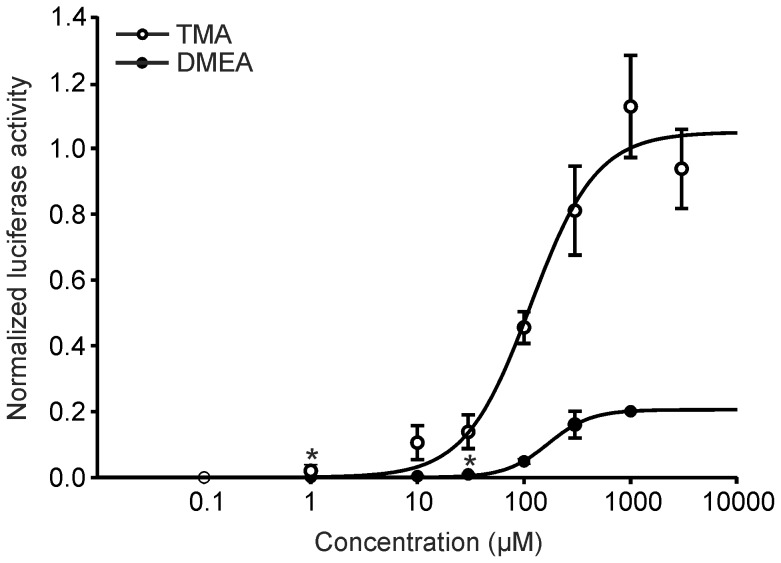
TMA and DMEA act as agonists at the hTAAR5 receptor in a concentration-dependent manner. Human TAAR5 responses were normalized to the response to forskolin (10 µM). EC_50_ = 116 µM (TMA) and EC_50_ = 169 µM (DMEA). Detection threshold for TMA is 1 µM (*p<0.05) and for DMEA 30 µM (*p<0.05). Error bars represent SEM.

In subsequent experiments we constructed concentration-response curves of the two active substances that revealed that 1 µM TMA is sufficient to induce significant signals above detection threshold (p<0.05). Adding of 1 mM TMA to the extracellular media led to the induction of a strong luciferase activity that was even higher than the signal induced by the adenylate cyclase activator forskolin (10 µM) as positive control. TMA is the most potent hTAAR5 ligand with an EC_50_ value of 116 µM (n = 2–13), followed by DMEA EC_50_ = 169 µM, n = 2–6) (Fig. 4). DMEA activates hTAAR5 with a lower efficacy and is therefore a partial agonist.

To compare the receptor affinities we additionally expressed mTAAR5 in HANA3A cells and measured receptor activity in the Cre-luciferase assay (Figure S2). The murine TAAR5 is more sensitive than the human ortholog. Calculated EC_50_ value is 940 nM (n = 2–5).

### Human TAAR5 Expression in *Xenopus laevis* Oocytes

**Figure 5:**
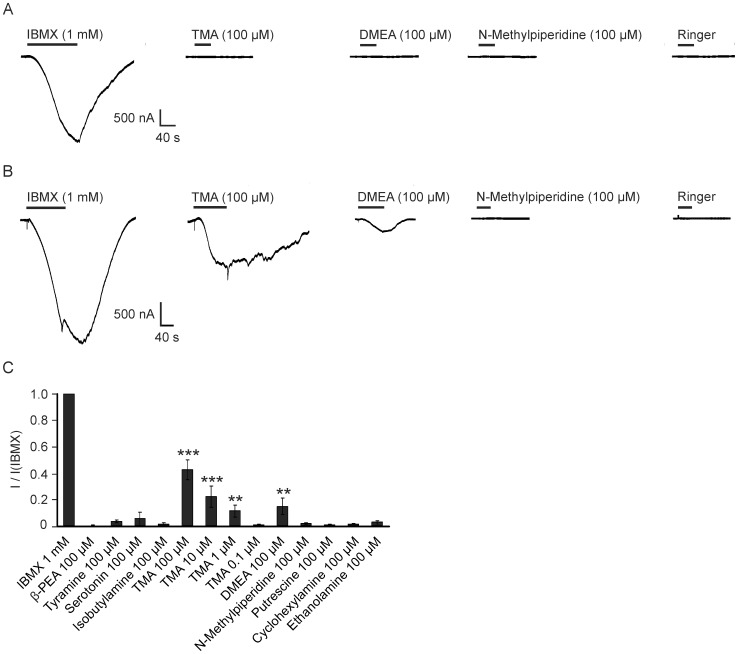
TMA and DMEA activate hTAAR5 expressed in *Xenopus* oocytes. A: IBMX induced currents in oocytes expressing the reporter channel CFTR only. **B:** In oocytes injected with hTAAR5 cRNA application of TMA and DMEA as well as IBMX as positive control generated currents. **C:** Test substance induced currents normalized to the corresponding IBMX response. TMA and DMEA induced currents significantly differing from Ringer control (***p<0.001; **p<0.01). Evoked currents were measured by two-electrode voltage clamp technique at a holding potential of −70 mM. Black bars indicate time of agonist application. Error bars represent SEM.

Due to the fact that co-expression of different proteins like RTP1S (Materials and methods) can alter the surface receptor expression and sensitivity of the used reporter system, EC_50_ values measured by only one expression system have limited reliabilities for statements about general receptor sensitivity. We used a different recombinant expression system to validate our data regarding the hTAAR5 sensitivity for the activating tertiary amines TMA and DMEA obtained by CRE-luciferase assay. We heterologously expressed hTAAR5 using *Xenopus laevis* oocytes, and screened hTAAR5 with various amines, focusing on DMEA and TMA. This system was used for h/mTAAR1 [1], [15] and mammalian odorant receptors and employs CFTR as a reporter channel [16], [17], necessary for the induction of currents (Materials and methods). As a control for CFTR expression level, each oocyte was tested for its sensitivity to the phosphodiesterase inhibitor isobutylmethylxantine (IBMX, 1 mM), which induces a rise in intracellular cAMP and subsequently CFTR mediated inward currents. Human TAAR5 was tested for a total of 10 different amines: β-phenylethylamine, tyramine, serotonin, isobutylamine, TMA, DMEA, N-methylpiperidine, putrescine, cyclohexylamine and ethanolamine, all applied at a concentration of 100 µM. TMA and DMEA induced inward currents on oocytes injected with hTAAR5 but failed to induce any currents in oocytes expressing the reporter channel only (Fig. 5A,B). Mean currents were higher for TMA (734±221 nA, n = 11) than for DMEA (136±56 nA, n = 6), both significantly smaller than the mean currents induced by IBMX (1625±19 nA, p<0.05, n = 15). The threshold of TMA detection was 1 µM (Fig. 5C), similar to the Cre-luciferase assay (Fig. 4). Normalized to the IBMX induced currents 100 µM, TMA and DMEA evoked 42.5±12.8% and 14.6±6.0% of the IBMX induced currents respectively (Fig. 5C). None of the other tested amines evoked notable currents. Our *Xenopus* data confirm that TMA and DMEA are activating ligands for human TAAR5.

### Characterization of SNPs in hTAAR Genes

Amoore identified TMA as the primary fishy odor and found that about 7% of the human population are specifically anosmic for this odorant [18]. For screening a large group of subjects (n = 393) with the primary odorant to find TMA anosmics, we used a standardized test concentration in water that is 16 times threshold [19]. In two different screenings with forced choice tests we identified 12 TMA anosmics. To figure out if the anosmia is caused by a SNP in an hTAAR coding sequence, especially in hTAAR5, the sequencing data from seven subjects were used for subsequent SNP analysis (see Materials and methods).

#### Reference data

On the Illumina GA_IIx_ platform, the exons of the six putatively functional human TAAR genes (hTAAR1, −2, −5, −6, −8 and −9) were sequenced in a pool of two hundred randomly selected subjects and scanned for putative Single Nucleotide Polymorphisms (SNPs). Allele frequencies of these putative SNPs were calculated using the CRISP algorithm [20]. In total, 12 SNPs were identified, with all of them having been annotated previously in dbSNP (Table 1). Additional allele frequency data based on the genotypes of 4,300 European American individuals was pulled from the NHLBI Grand Opportunity Exome Sequencing Project’s (ESP) Exome Variant Server, release ESP6500 [21]. In general, allele frequency estimates based on the control pool data aligned well with frequencies from the ESP dataset (Table 1).

**Table 1:** SNPs in the coding regions of hTAAR genes.

		SNP Position		Nucleotide		SNP Frequency			
Gene	SNP ID	on Chr. 6	in mRNA	Reference	SNP	ESP Cohort	Ctrl Pool	TMA Anos.	p-value (FET)
TAAR1	rs8192620	132966279	864	T	C	22.9	30.2	28.6	1.000
	rs8192619	132966348	795	G	A	5.4	4.7	7.1	0.506
TAAR2	rs61745666	132938817	528	G	A	1.9	3.1	0.0	1.000
	rs8192646	132938842	503	C	T	2.2	5.6	0.0	1.000
TAAR5	rs3813355	132910612	266	G	A	65.2	66.2	71.4	0.781
	rs3813354	132910634	244	C	T	9.4	10.8	7.1	1.000
TAAR6	rs8192622	132891538	78	C	T	4.9	6.4	0.0	1.000
	rs8192624	132892253	793	G	A	8.0	6.7	7.1	1.000
	rs8192625	132892332	872	G	A	6.8	6.5	14.3	0.243
TAAR8	rs8192627	132874814	983	A	C	7.2	5.8	0.0	1.000
TAAR9	rs2842899	132859609	181	A	T	27.0	24.6	35.7	0.351
	rs9402420	132859437	11	C	T	9.2	6.5	0.0	1.000

Calculation of Fisher’s Exact Test based on SNP percentage difference between control pool and TMA anosmics.

#### Analyzing hTAAR genes from TMA anosmics

A second round of sequencing included seven subjects with a specific anosmia for trimethylamine (TMA). The coding regions of the six putatively functional human TAAR genes (hTAAR1, −2, −5, −6, −8 and −9) were sequenced individually by Sanger sequencing. In total, seven SNPs were found in at least one of the anosmic subjects (Table 1). All of the identified SNPs were also present in the control pool. In the hTAAR5 gene, two SNPs were found. However, both of them are synonymous and do not occur at a significantly different frequency in the anosmics’ group compared to the control pool (Fisher’s Exact Test, p>0.05). A loss-of-function variant in the hTAAR9 gene (rs2842899) was more frequent in the group of anosmics than in the control pool, but not significantly (p = 0.351, Fisher’s Exact Test).

Five SNPs that had been identified in the control pool were not found in the group of anosmics, but the differences in their allele frequencies (assumed to be 0% for the anosmics) were not statistically significant. The same is true for four SNPs found in the hTAAR1 and hTAAR6 genes. These data show that there is no association between a polymorphism in an hTAAR coding sequence and the observed anosmic individuals.

## Discussion

In our study, we present the first deorphanization of a human olfactory TAAR. Human TAAR5 can be activated with a high specificity by the tertiary amines TMA (full agonist) and DMEA (partial agonist), but not by any other of the tested aminic compounds. Interestingly, TAAR5 ligand specificity differs between humans and mice. The human receptor is exclusively activated by tertiary amines with methyl or ethyl side chains. In contrast, the murine TAAR5 ortholog is also activated by N-methylpiperidine and the secondary amine dimethylamine [2], [11].

The finding that TMA activates the hTAAR5 receptor is of substantial interest because this receptor is the most highly expressed TAAR gene in the human OE [12], [22]. It is still unknown whether human TAARs are expressed in OSNs; however, hTAAR5 expression was detected exclusively in OMP-positive biopsy probes, which indicates that this TAAR is expressed specifically in the OE. In humans, TMA is a product of the choline metabolism and is decomposed by N-oxygenation into the odorless trimethylamine N-oxide (TMAO), which is excreted via urine. If the body loses the ability to decompose TMA, then TMA with its unpleasant odor of rotting fish will be released in bodily secretions like sweat, breath and urine. This genetic disease is called Trimethylaminuria (TMAU) [23], [24]. Furthermore, TMA arises in rotting male ejaculate and vaginal secretions. TMA levels may be regulated by sexual hormones [25], and cycle-dependent threshold variations were reported in women [26]. TMA may be a social odor in humans or a scent of disease [24]. Two recent studies dealing with the axonal projections to the mouse olfactory bulb show that TAAR expressing OSNs project to glomeruli close to domain I and II. The authors conclude that TAARs constitute an olfactory subsystem detecting volatile amines that elicit innate behavior or physiological responses, at least in mice [27], [28]. For a long time it has been controversially discussed if there are pheromone-like substances that have distinct effects on human behavior.

Using the Cre-SEAP assay system, Stäubert et al. (2010) showed that TAAR5 of primates including humans could not be activated by TMA. This contradictionary finding may be explained by differences in the sensitivity of the assay systems. We used the Cre-luciferase assay system. Measurements revealed a detection threshold of 1 µM and an EC_50_ value of 116 µM for the human TAAR5, while the EC_50_ for murine TAAR5 was previously reported to be nearly 400-fold lower (300 nM) [2]. Due to the different assay sensitivities the comparability of the EC_50_ values is somehow limited. Therefore, we tested the murine TAAR5 receptor activity and determined an EC_50_ value of 940 nM under the same Cre-luciferase assay conditions we used for human TAAR5 (Figure S2). This confirms that the murine TAAR5 is more sensitive than the human ortholog, at least in a recombinant system. However, it could still play an important role within human olfaction.

In a recombinant system the co-expression of different proteins like REEPs or RTPs can influence the receptor cell-surface expression [14], [29], which essentially determines measured intensities of receptor activation. We co-transfected RTP1S and G_olf_ that might increase the surface expression of m/hTAAR5 and general assay sensitivity, but there might be even more optimized expression conditions for each receptor. It is also possible that receptors expressed *in vivo* in OSNs are more sensitive than receptors expressed *in vitro* in a recombinant system. The olfactory detection threshold for TMA in water is 4.7×10^−7^ g/l, which is equivalent to 8 nM [30]. In a recombinant system, even the sensitive murine TAAR5 is not activated by such a low TMA concentration. The low olfactory detection threshold for TMA is similar to that for 5α-androst-16-en-3-one, a human steroid in male and female urine and sweat [30]. *In vitro*, the olfactory receptor OR7D4 is selectively activated by androstenone with an EC_50_ value of 12 µM, which is also above the olfactory threshold concentration [31]. It seems to be not quite clear to what extent receptor sensitivities in recombinant systems can be transferred to *in vivo* situations, where the receptor is expressed in native OSNs. Nevertheless, the general functionality can be tested. Furthermore, there is a link between the function of OR7D4 *in vitro* and the rating of androstenone *in vivo* [31], as well as between the function of OR11H7P *in vitro* and threshold variations in the perception of isovaleric acid *in vivo* [32].

In both cases, SNPs in the coding sequence of odorant receptors were responsible for phenotypic variations. Many odor-specific anosmias are known, although their molecular background remains enigmatic. Thus, we investigated whether any SNP in a functional hTAAR gene was associated with TMA anosmia and compared the determined SNP frequency with that found in a Caucasian control group. No significant association was found in any of the hTAAR coding sequences. Interestingly, no non-synonymous SNP in the coding sequence of hTAAR5 with a frequency greater than 2.8% has been reported (dbSNP build 135). However, assuming that solely a single polymorphism in the TMA receptor gene TAAR5 is responsible for the specific anosmia for TMA present in 7% of the population [18], the frequency of the causative loss-of-function allele would be expected to be 26.5% for a recessive disorder and 3.6% for a dominant disorder, as long as the population is in Hardy-Weinberg equilibrium. Therefore, we propose the molecular reason for the observed TMA anosmia is independent of a mutation within the hTAAR5 coding sequence. Due to the fact that we focused on analyzing the hTAAR reading frames, it is possible that there is a molecular reason we did not identify, because the mutation may be elsewhere in the hTAAR5 gene or in a gene regulator element. We cannot exclude the presence of a mutation within the coding sequence of another high-affinity TMA sensor responsible for TMA anosmia. To identify the TMA anosmics, we used a standardized test concentration that is 16 times higher than the olfactory detection threshold [19]. Amoore used also higher TMA concentrations and showed that the average specific anosmic can barely detect a TMA concentration that is 830 times the detection threshold in water [30]. It might be that human TAAR5 is activated only by higher TMA concentrations. Higher TMA concentrations may occur in specific human physiological or pathophysiological situations. In a very recent study, Li et al. suggested the existence of additional TMA receptors as well. They showed that TAAR5 is required for species-specific behavior of mice smelling TMA present in mouse urine. Murine TAAR5 knockout indeed abolished the attraction to TMA, but retained avoidance behavior to higher TMA concentrations [33].

In the end, it still remains elusive which receptors are involved in the perception of TMA and if TAARs mediate physiological responses via an amine-specific olfactory subsystem in humans.

### Conclusion

Since the identification of TAARs as a second class of olfactory receptors in the OE of vertebrates in the last decade, we have been able to show for the first time that human “olfactory” TAARs can be functional in a recombinant expression system. Human TAAR5 is specifically activated by TMA, a highly volatile aminic compound and the prototype of fishy odor. Thus, it imperatively stands to reason that also human TAAR orthologs can be functional *in vivo* and might be a molecular sensor for the detection of volatile amines. Moreover, as TMA occurs in bodily secretions, human TAAR receptors could revive the olfactory research of human social cues.

## Materials and Methods

### Ethics Statement

Experiments were approved by the ethics committee of the University Hospital of the “Technische Universität Dresden” (No. EK40022009). We had consent in both, verbal and writing. Subjects also received a copy of the information sheet and of the consent form. The consent form was also signed by the investigator. The ethics committee approved the consent procedure.

### Expression Plasmids

TAAR expression vector similar to those for rho-tagged odorant receptors [34] coding for a rho-tagged hTAAR5 in the pCI-vector as well as expression vectors for CFTR in pSGEM and mRTP1S in pCDNA3 were constructed by standard PCR methods. Plasmids containing G_olf_, RTP1, RTP2 and REEP1 were a kind gift from C.W. Lütje and H. Matsunami [17], [29]. Cloned Ric8b was donated by B. Malnic [35]. For the reporter gene assay, pGL4-luciferase and pRL-TK-*Renilla* (Promega) were used.

### Immunocytochemistry and CRE-luciferase Assay

We thank H. Matsunami (Duke University Medical Center, Durham, N.C.) for the kind donation of HANA3A cells [29]. For immunocytochemical proving of the transfection efficiency and the expression of the hTAAR5 protein in transfected HANA3A cells respectively, we performed a fixation step for 20 min in 4% paraformaldehyde at 4°C. Then, the monoclonal anti-rhodopsin antibody 4D2 (Mobitec) was applied for 2 h at RT. After a washing step, the secondary antibody coupled to Alexa Fluor 488 (Invitrogen) was applied for 45 min at RT. Immunocytochemical evaluation of the hTAAR5 cell-surface expression was done by live-cell staining, according to the protocol of Zhuang and Matsunami 2008. Pictures were taken with a Zeiss confocal microscope (LSM510 Meta; Zeiss).

For a functional assay, we adapted the CRE-luciferase system optimized for odorant receptor screening by Zhuang and Matsunami (2008). HANA3A cells were maintained under standard conditions in DMEM supplemented with 10% FBS, 100 units/ml penicillin and streptomycin at 37°C. Cells were plated on poly-D-lysine–coated 96-well plates (NUNC) 1 day before the assay (about 15,000 cells/well) and transfected with Lipofectamine 2000 (Invitrogen) according to the manufacturer’s protocol using 18 µl Lipofectamine, 5 µg hTAAR5, 1 µg RTP1S, 0.5 µg G_olf_, 2 µg pGL4-luciferase and 1 µg pRL-TK-*Renilla* plasmid for a complete 96-well plate.

Eighteen to 24 hours after transfection, cells were stimulated with agonists diluted in CD293 with 2 mM L-glutamine for 4 hours at 37°C. The Dual-Glo Luciferase Assay System (Promega) was used to measure the activation of the transfected TAARs. *Renilla* luciferase driven by a constitutively active TK-promoter (pRL-TK-*Renilla*) served as an internal control to determine cell viability and transfection efficiency. We normalized firefly luciferase activity with the formula (Luc/Ren(N) – Luc/Ren(min))/(Luc/Ren(max) – Luc/Ren(min)), where Luc/Ren(N) is the luminescence of firefly luciferase divided by luminescence of *Renilla* luciferase in a certain well. L_min_ is the minimum luciferase ratio of TAAR transfected cells to Ringer control on a plate, and L_max_ is the maximum luciferase ratio of TAAR transfected cells to forskolin control on a plate. Mock-transfected cells were stimulated to exclude unspecific responses to the tested substances.

### Functional Expression of Receptor cRNA in *Xenopus* Oocytes

Expression of cRNA and electrophysiological experiments were essentially performed as described [36]. cRNA was synthesized using the AmpliCap T7/T3 High Yield Message Maker Kit (Epicenter, Madison, WI), according to the manufacturer’s protocol, with linearized plasmids as templates. *Xenopus laevis* oocytes were prepared by collagenase digestion. After 24 h, stage IV–VI oocytes were injected with cRNA (typically 24 ng/oocyte: a mix of 10 ng TAAR5, 5 ng CFTR, 5 ng G_olf_, 1 ng RTP1, 1 ng RTP2, 1 ng REEP1 and 1 ng Ric8b cRNA) and incubated at 18°C in Barth’s solution. Two-electrode voltage clamp recordings were generated after 2–4 days at room temperature. Agonists were diluted to the concentrations indicated with Frog-Ringer’s solution (115 mM NaCl, 2.5 mM KCl, 1.8 mM CaCl_2_, 10 mM HEPES, pH 7.2). Recording was done with a two-electrode voltage clamp amplifier (TURBO TEC-03, npi, Tamm, Germany) and pCLAMP software (Axon Instruments, Union City, CA) with typical holding potential of −70 mV. 1 mM 3-isobuthyl-1-methylxanthline (IBMX) induced currents served as a control for expression of the reporter gene cystic fibrosis transmembrane conductance regulator (CFTR). Initial data analysis was done by Clampfit software (Molecular Devices).

### Screening for TMA Anosmics and Genotyping

TMA anosmics were identified by a forced choice test and solutions for TMA testing were essentially prepared as described by Amoore [19]. Healthy students (age 20–30) were challenged with TMA and two blank probes. Initially identified anosmics were those who failed to recognize TMA two times in a forced choice test. They were retested after 1–7 days and only anosmics in both tests were considered for further experiments. All TMA anosmics were able to smell pyridine in a concentration of 68 ppm (parts per million) as a general control. In total, we screened 393 volunteers and identified 12 subjects with specific anosmia confirmed in repeated tests. The identified fraction is smaller than the 7% expected [18], caused by the fact that not all initially found anosmics could be retested.

Genomic DNA was obtained from the identified TMA anosmics and prepared from buccal swabs with my-Budget Saliva DNA Kit (Bio-Budget Technologies GmbH, Krefeld, Germany) according to the manufacturer’s instructions. TAAR gene sequences were enriched by PCR, using gene specific primers located directly in front of and behind the protein-coding sequence, and purified by agarose gel electrophoresis followed by extraction with the Wizard SV Gel and PCR Clean-Up System (Promega Corporation, Madison, Wisconsin). Purified PCR products were then sequenced by the Ruhr-University Bochum sequencing service using the Applied Biosystems 3130×l Genetic Analyzer sequencing setup. Fluorescence flow diagram outputs were viewed and analyzed with the DNASTAR Lasergene SeqMan software package.

### Illumina Sequencing of Control Pool

Of the 393 subjects that had been screened for a TMA anosmia, 200 were selected at random to make up the control pool. Their DNA was prepared from buccal swabs using the my-Budget Saliva DNA Kit. DNA concentrations were determined by OD measurement with the NanoDrop ND-1000 spectrophotometer (PEQLAB Biotechnologie GmbH, Erlangen, Germany). Equal amounts (µg) of individuals’ DNAs were pooled into a single sample. From the pooled sample, sequences for the six functional human TAAR genes were enriched by PCR and products were purified by agarose gel electrophoresis and subsequently extracted with the Wizard SV Gel and PCR Clean-Up System. Purified PCR products were pooled into a single sample, which was given to the Cologne Center for Genomics NGS unit, where it was sequenced on the Illumina GA_IIx_ sequencing platform. Raw sequence data was aligned to the human genome reference sequence (hg19) using the bowtie algorithm [37] with the “–best”-parameter. Output BAM-files were sorted and indexed using the SAMtools software package [38]. Alignments were then scanned for SNPs with the CRISP algorithm (Comprehensive Read analysis for Identification of Single Nucleotide Polymorphisms from Pooled sequencing [20]), using the algorithm’s default parameters for SNP calls. Analysis of the reported SNP frequencies was performed with Microsoft Excel 2010 and R version 2.11.1.

### Oligonucleotides

1. hTAAR1_fwd: GCGCGGCCGCACCATGATGCCCTTTTGCCACAATATAATTAATAT hTAAR1_rv: GCGGCGGCCGCTGAACTCAATTCCAAAAATAATTTACACC
2. hTAAR2_fwd: GCATATGAATTCATGTATTCATTTATGGCAGGAT hTAAR2_rv: GCATATGCGGCCGCCTACTCACTTTCTTTTTGCATACAC
3. hTAAR5_fwd: GCTATCTATGCTGCATTTGATTTTCAGG hTAAR5_rv: GCTATCATTGAATGTGGGGAGTGCT
4. hTAAR6_fwd: GCATATGAATTCATGAGCAGCAATTCATCCCTGC hTAAR6_rv: GCATATGCGGCCGCTTATATATGTTCAGAAAACAAATTCATG
5. hTAAR8_fwd: GCATATGAATTCATGACCAGCAATTTTTCCCA hTAAR8_rv: GCATATGCGGCCGCTTATTCTAAAAATAAACTAATGGTTGATGA
6. two overlapping fragments hTAAR9_fwd1: CAGAAGATAAACTAACACACAAGA hTAAR9_rv1: GCATATGCGGCCGCAATAAATTAGTTGTTGACGAATCAGT hTAAR9_fwd2: GCATATGAATTCATGGTGAACAATTTCTCCCAAG hTAAR9_rv2: GCTATCAGTAATGTTTTTAATCTGTCTCTACTTCTTC

### Statistics

For electrophysiological measurements and reporter gene assays, statistical analysis and curve fitting was done by the Hill equation using Microsoft Excel 2010 or SigmaPlot V8.0 (Systat Software, San Jose, CA). Error bars represent SEM.

### Chemicals

All tested aminergic substances and standard chemicals were from Sigma Aldrich or J.T. Baker and dissolved as 100 mM stocks in Frog-Ringer`s solution with the exception of odorants in DMSO. Chemicals used for the initial screening of hTAAR5 were: trimethylamine, tetramethylammonium hydroxide, triethylamine, triethanolamine, tetraethylammonium chloride, β-phenylethylamine, methylamine, dimethylamine, dimethylbutylamine, dimethylethylamine, dimethylisopropylamine, taurine, diethylmethylamine, tyramine, trimethylamine N-oxide, carnosine, octopamine, γ-aminobutyric acid, histamine, cyclohexylamine, serotonin, ethanolamine, acetylcholine, choline, aminoacetophenone, N-methylpiperidine, isobutylamine, tropine, pyridine, piperine, tropane, methylmorpholine, dimethylpyrazine, 2-isobutyl-3-methoxypyrazine, (−)-nicotine, (+)-nicotine, cotinine, trimethylphosphine, skatole (3-methylindole), putrescine (1,4-diaminobutane), spermidine, cadaverine, muscone, ω-pentadecalactone, globalide, galaxolide, aurelione, and Henkel 100 (Henkel, Düsseldorf, Germany) [16].

## Supporting Information

Figure S1 **Evaluation of cell-surface hTAAR5 receptor expression.** Expression of the rhodopsin-tagged hTAAR5 receptor in transfected HANA3A cells was detected by immunocytochemical live-cell staining, using the anti-rhodopsin antibody 4D2 and a secondary antibody labeled with the fluorescent dye Alexa Fluor 488 (green). Cell nuclei were stained by DAPI (blue). Scaling bar: 10 µm.
(TIF)

Figure S2 **Concentration response curve of mTAAR5.** Responses to TMA were normalized to the response to forskolin (10 µM). Calculated EC_50_ for mTAAR5 is 940 nM. At the same time we repeated measurements for hTAAR5 and were able to reproduce previously calculated EC_50_ around 100 µM (n = 4). Data are given as mean ± SEM of 2–5 independent experiments, each performed in duplicates. Error bars represent SEM.
(TIF)
