# Supplementary figures and images for: Human Trace Amine-Associated Receptor TAAR5 Can Be Activated by Trimethylamine
Source: PLoS One. 2013 Feb 5;8(2):e54950. doi: 10.1371/journal.pone.0054950 (PMC3564852; doi:10.1371/journal.pone.0054950)

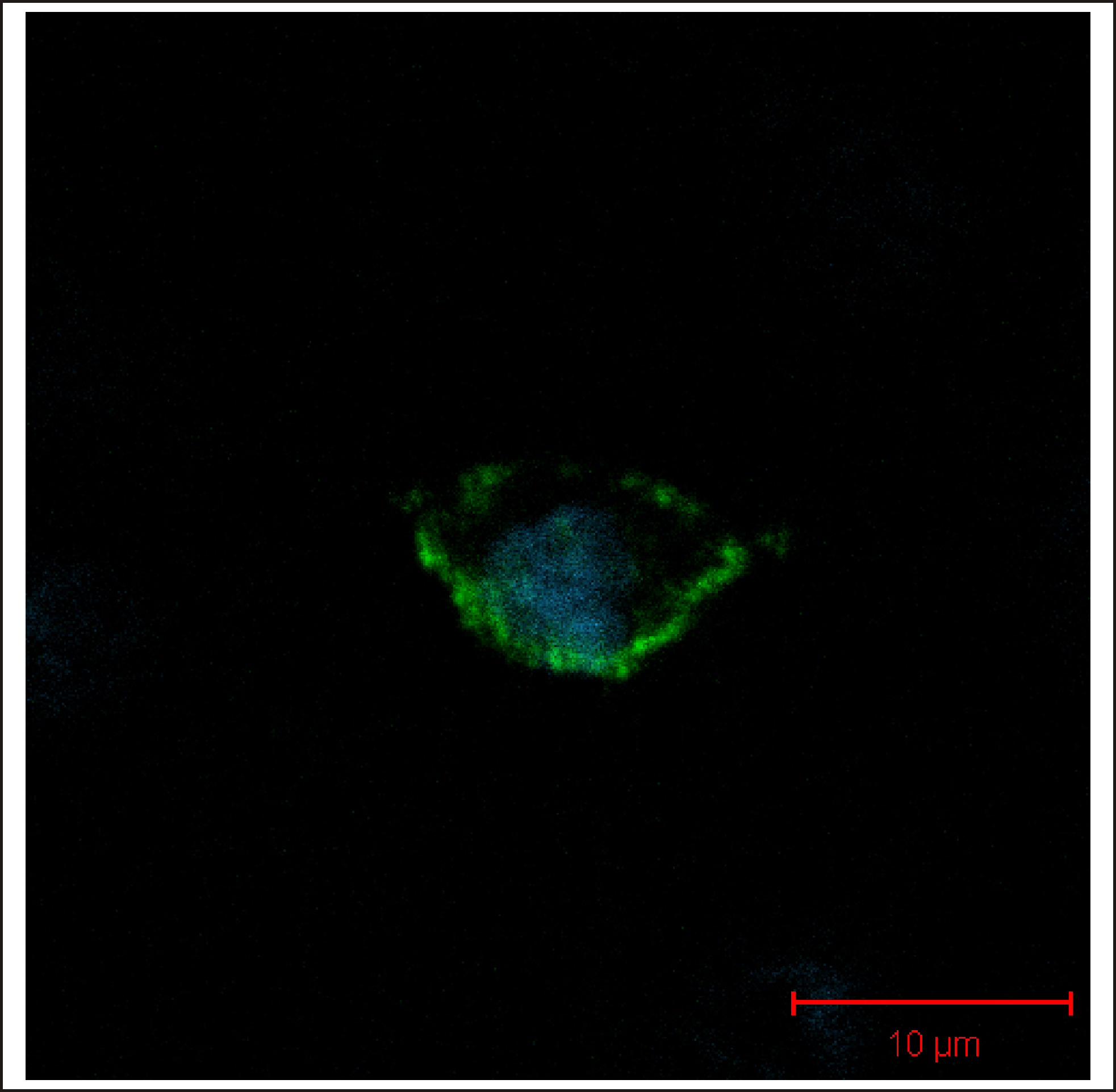

Supplement: Figure S1 — Evaluation of cell-surface hTAAR5 receptor expression. Expression of the rhodopsin-tagged hTAAR5 receptor in transfected HANA3A cells was detected by immunocytochemical live-cell staining, using the anti-rhodopsin antibody 4D2 and a secondary antibody labeled with the fluorescent dye Alexa Fluor 488 (green). Cell nuclei were stained by DAPI (blue). Scaling bar: 10 µm. (TIF) [file pone.0054950.s001.tif]

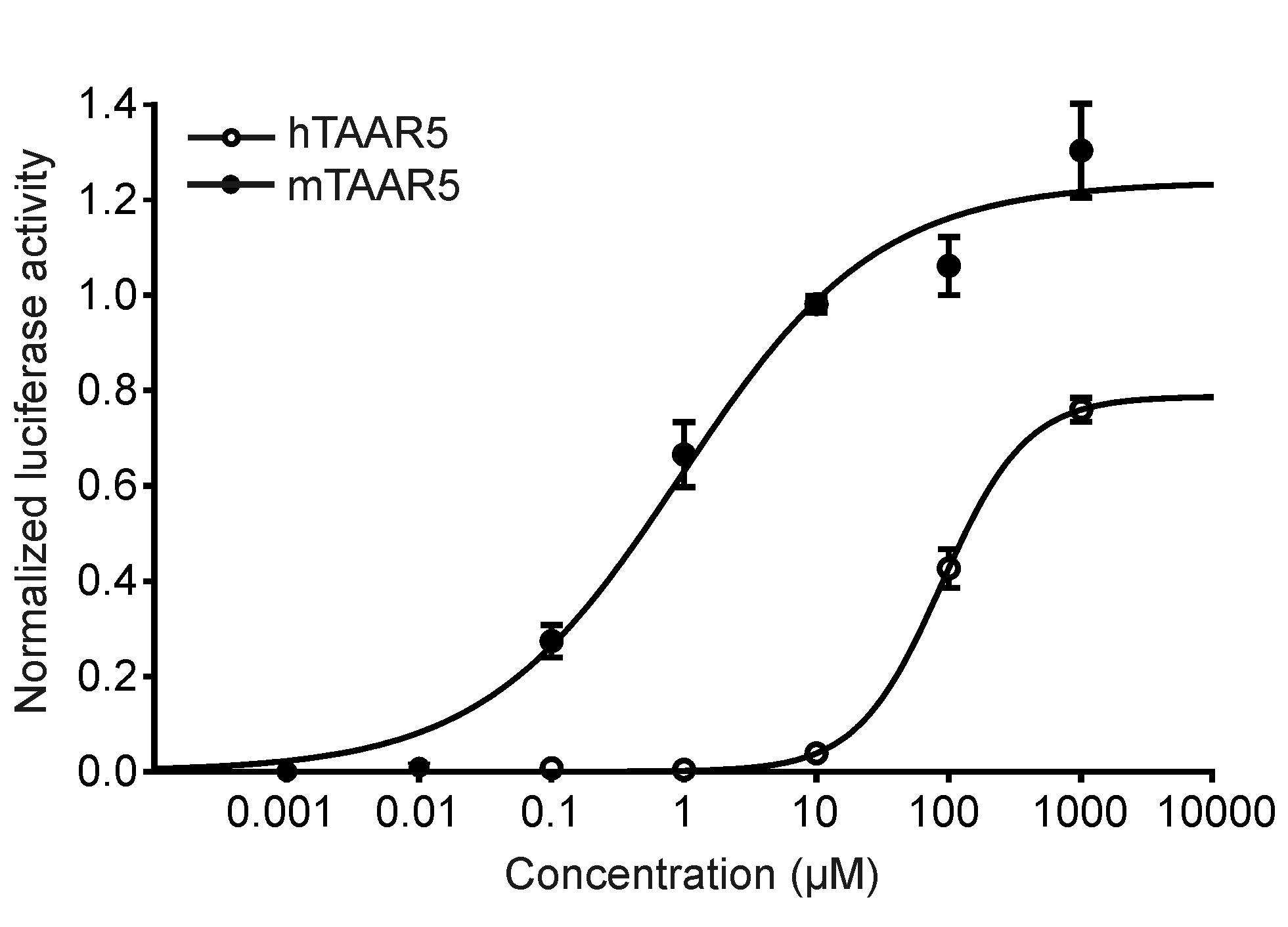

Supplement: Figure S2 — Concentration response curve of mTAAR5. Responses to TMA were normalized to the response to forskolin (10 µM). Calculated EC50 for mTAAR5 is 940 nM. At the same time we repeated measurements for hTAAR5 and were able to reproduce previously calculated EC50 around 100 µM (n = 4). Data are given as mean ± SEM of 2–5 independent experiments, each performed in duplicates. Error bars represent SEM. (TIF) [file pone.0054950.s002.tif]
